# Different Selective Effects on Rhizosphere Bacteria Exerted by Genetically Modified versus Conventional Potato Lines

**DOI:** 10.1371/journal.pone.0067948

**Published:** 2013-07-03

**Authors:** Armando Cavalcante Franco Dias, Francisco Dini-Andreote, Silja Emilia Hannula, Fernando Dini Andreote, Michele de Cássia Pereira e Silva, Joana Falcão Salles, Wietse de Boer, Johannes van Veen, Jan Dirk van Elsas

**Affiliations:** 1 Department of Microbial Ecology, Centre for Ecological and Evolutionary Studies (CEES), University of Groningen (RUG), Groningen, The Netherlands; 2 Department of Microbial Ecology, Netherlands Institute of Ecology (NIOO-KNAW), Wageningen, The Netherlands; 3 Department of Soil Science, ESALQ/USP, University of São Paulo, Piracicaba, Brazil; Wageningen University and Research Centre, The Netherlands

## Abstract

**Background:**

In this study, we assessed the actively metabolizing bacteria in the rhizosphere of potato using two potato cultivars, i.e. the genetically-modified (GM) cultivar Modena (having tubers with altered starch content) and the near-isogenic non-GM cultivar Karnico. To achieve our aims, we pulse-labelled plants at EC90 stage with ^13^C-CO_2_ and analysed their rhizosphere microbial communities 24 h, 5 and 12 days following the pulse. In the analyses, phospholipid fatty acid/stable isotope probing (PLFA-SIP) as well as RNA-SIP followed by reverse transcription and PCR-DGGE and clone library analysis, were used to determine the bacterial groups that actively respond to the root-released ^13^C labelled carbonaceous compounds.

**Methodology/Principal findings:**

The PLFA-SIP data revealed major roles of bacteria in the uptake of root-released ^13^C carbon, which grossly increased with time. Gram-negative bacteria, including members of the genera *Pseudomonas* and *Burkholderia,* were strong accumulators of the ^13^C-labeled compounds at the two cultivars, whereas Gram-positive bacteria were lesser responders. PCR-DGGE analysis of cDNA produced from the two cultivar types showed that these had selected different bacterial, alpha- and betaproteobacterial communities at all time points. Moreover, an effect of time was observed, indicating dynamism in the structure of the active bacterial communities. PCR-DGGE as well as clone library analyses revealed that the main bacterial responders at cultivar Karnico were taxonomically affiliated with the genus *Pseudomonas,* next to *Gluconacetobacter* and *Paracoccus*. Cultivar Modena mainly attracted *Burkholderia*, next to *Moraxella*-like (Moraxellaceae family) and *Sphingomonas* types.

**Conclusions/Significance:**

Based on the use of *Pseudomonas* and *Burkholderia* as proxies for differentially-selected bacterial genera, we conclude that the selective forces exerted by potato cultivar Modena on the active bacterial populations differed from those exerted by cultivar Karnico.

## Introduction

The living soil is often grossly carbon-limited and this constraint poses severe restrictions to the growth of heterotrophic bacteria. On the other hand, the rhizosphere of plants represents a hotspot in soil where organic carbonaceous compounds are released by the roots [1]. There is compelling evidence for the statement that such compounds act as a sophisticated interplay of gradient-wise chemical signalling and nutrition, resulting in a core suite of microbes which are able to successfully compete and thrive at the roots [2]. Thus, plants act as selectors of particular soil bacteria into their rhizospheres, promoting root colonization by primary and even secondary responders to root-excreted compounds [3], [4]. Given the fact that rhizosphere microorganisms interact with the plant roots as well as with local phytopathogens, the structure of the microbial community that is established in the rhizosphere has a strong bearing on plant functioning in terms of growth and health [5].

Genetically modified (GM) plants are important to agriculture, as they can offer several key benefits to agricultural practices, including yield increases. In potato, GM derivatives have been produced that allocate different amounts of amylose/amylopectin to the tubers [6]. Thus, cultivar Modena, derived from parental cultivar Karnico, produces tubers with altered starch content. However, the use of GM crops has raised a number of concerns about their potential impact on soil ecosystems [7], [8]. Specifically in the Modena event, altered root exudation patterns may have resulted from the genetic modification [9], [10]. Such altered root exudation possibly exerts an effect on the selection of bacterial communities at the roots.

In plant-soil systems, CO_2_ is the main source of carbon that, following photosynthesis, ends up in the plant root released compounds in the soil and then flows into the microorganisms in the rhizosphere [11], [12]. Up to 50% of the total carbon fixed by photosynthesis is indeed transferred to the roots. From this total, approximately half is further released into the soil [13]. This amazing amount of fixed carbon is likely to be captured by those (heterotrophic) rhizosphere microorganisms that have evolved the capacity to rapidly respond. However, in spite of their importance for plant health and growth, we still lack information about such root-activated organisms and their ecology in the rhizosphere.

Stable isotope probing (SIP), on the basis of the ^13^C isotope, combined with tools for molecular detection, is a very suitable method to track the allocation of plant root released carbon into rhizosphere microorganisms [14], [15], [16]. In recent years, SIP has been applied to characterize microorganisms that capture plant root exudates in peat land, grasses, *Arabidopsis thaliana* [17], [18], wheat, maize, rape and barrel clove [19]. Moreover, it has been used to distinguish the bacterial groups that are actively involved in specific biogeochemical processes at rice roots [20] in a grassland soil [21] and living as endophytes in ^13^C-enriched potato plants [22].

In this study, using both PLFA-SIP and RNA-SIP approaches, we examined the bacterial communities that effectively accumulate the ^13^C label in carbonaceous molecules that are released by the roots of two different potato cultivars, i.e. the ware potato Karnico and its GM derivative Modena, into the rhizosphere soil system. Our hypotheses were (1) that particular subsets of the total bacterial communities are selected by the carbonaceous compounds released from the roots, and (2) that such responses are different between the near-isogenic parent plant and its GM derivative.

## Materials and Methods

### Ethic Statement

No specific permits were required for the described field studies. The locations are not protected. The field studies did not involve endangered or protected species.

### Experimental Set-up, 13C Labelling and Harvesting

This work accompanies the recent study by Hannula *et al.* [23], in which the active fungal communities in the rhizosphere of senescent potato plants were examined. Briefly, the experiment compared the communities at the GM potato (*Solanum tuberosum* L.) line Modena (with altered tuber starch quality of use for industrial purposes) with those of its parental line Karnico. The soil used for the experiments was a sandy peat soil collected from a Dutch agricultural field (for more details see [23], [24]). The soil was homogenized and sieved (<2 mm), after which it was transferred to sterilized pots (volume of 10 L). One tuber of either cultivar was planted per pot and the plants were grown in the greenhouse. After robust plants had formed, these were labelled for a total of 24 h, by exposing them to an atmosphere of ^13^C-CO_2_ at atmospheric partial pressure inside special chambers. Control plants were exposed to ^12^C-CO_2_. After the labelling period, pots were removed from the chambers and the rhizosphere soil of three replicate plants per cultivar was harvested from both the ^13^CO_2_ and ^12^CO_2_ treatments. The samples were collected in three distinct periods after the CO_2_ pulse: i.e. 24 h, 5 and 12 days. For more details, see [23]. Concerning the rhizosphere sampling, only the soil that adhered strongly to the plant roots was considered to compose the rhizosphere. Thus, we avoided to a maximum extent the inclusion of bulk soil in the sample sets.

### Phospholipid Fatty Acid (PLFA) Analyses

PLFAs were extracted from rhizosphere soil samples using standard procedures [23]. The PLFA concentrations and δ^13^C values were then measured on a Finnigan Delta-S gas chromatograph–isotope ratio monitoring mass spectrometer (GC-IRMS), as in Boschker [25]. The internal standard methyl nonadecanoate fatty acid (19∶0) was used for calculating the concentrations. As biomarkers for bacteria, we used the following fatty acids: i14∶0, i15∶0, a15∶0, i16∶0, 16∶1ω7t, 17∶1ω7, a17∶1ω7, i17∶0, cy17∶0, 18∶1ω7c, cy19∶0 and 10 Me16∶0 [26]. The PLFAs i15∶0, a15∶0, i16∶0, i17∶0 and 10 Me16∶0, which are found mainly in Gram-positive bacteria, and 16∶1ω7t, 18∶1ω7c, cy17∶0 and cy19∶0 (Gram-negative bacteria), were used as indicators for these specific bacterial groups [27], whereas the cyclopropyl PLFAs cy17∶0 and cy19∶0 were used as biomarkers for *Pseudomonas* and *Burkholderia*, respectively [27]. PLFA 10 Me16∶0 was used as a specific indicator for *Actinobacteria* [28], PLFA 16∶1×5 mainly for arbuscular mycorrhizal fungi (AMF) and PLFA 20∶4ω6 for protozoan biomass [26] (Table 1). The percentage of ^13^C allocated to a certain PLFA was calculated from the amount of ^13^C in each PLFA compared to the total ^13^C accumulation (excess ^13^C pmol g^−1^) in all PLFAs used as biomarkers for different microbial groups, and these values were used in data analyses.

**Table 1:** Distribution of accumulated ^13^C in microbial PLFAs in the rhizosphere of pulse-labelled potato plants.

Bacteria	PFLA's	24 hours		5 days		12 days	
		Karnico	Modena	Karnico	Modena	Karnico	Modena
***Gram-positive***							
	i15∶0	1.45±0.1	1.24±0.04	3.32±0.17	2.28±0.31	1.85±0.50	0.43±1.41
	a15∶0	0.09±0.01	0.07±0.02	1.10±0.20	0.94±0.14	0.99±0.09	0.21±1.25
	i16∶0	0.06±0.01	0.10±0.03	0.63±0.02	0.70±0.16	0.69±0.05	0.11±0.05
*Bacillus*	i17∶0	3.42±0.53	2.01±0.20	1.91±0.02	1.08±0.29	4.38±1.11	3.23±0.37
*Actinobacteria*	10 Me16∶0	0.38±0.09	0.00±0.00	1.72±0.32	4.95±1.91	1.70±0.51	3.02±0.35
***Total Gram-positive***		**5.40±0.47**	**3.42±0.29**	**8.68±0.26**	**9.95±0.58**	**9.61±0.53**	**7.20±0.52**
***Gram-negative***							
	16∶1ω7t	2.70±0.40	5.39±0.59	4.46±0.77	3.99±3.26	5.65±0.92	1.65±0.41
	18∶1ω7c	4.38±1.89	7.94±1.07	30.57±3.05	18.23±0.89	10.73±1.29	33.30±2.98
*Pseudomonas*	cy17∶0	0.08±0.03	0.18±0.02	5.06±0.69	4.47±0.82	2.18±0.27	0.54±0.75
*Burkholderia*	cy19∶0	0.00±0.00	0.14±0.08	0.00±0.00	0.00±0.00	0.78±0.16	3.30±0.39
***Total Gram-negative***		**7.16±0.80**	**13.65±1.14**	**40.09±3.70**	**29.69±2.78**	**19.35±1.38**	**38.79±2.67**
***Non-specific bacteria PFLA***							
	17∶1ω7	0.70±0.07	0.19±0.03	2.80±0.08	7.01±0.63	2.37±0.31	4.41±0.52
	a17∶1ω7	0.19±0.07	0.00±0.00	0.93±0.13	2.54±0.22	0.89±0.05	1.30±0.55
	i14∶0	0.02±0.00	0.04±0.00	0.19±0.03	0.22±0.04	0.12±0.04	0.48±0.04
***Total Bacteria***		**13.23±1.21**	**17.14±1.77**	**47.36±5.37**	**41.84±5.06**	**30.21±2.81**	**57.66±4.39**
***Fungi***	18∶2ω6.9	78.68±2.69	75.77±1.03	33.48±0.98	39.62±1.77	63.30±3.78	28.29±4.33
***AMF***	16∶1ω5	7.43±1.71	6.90±0.29	13.73±1.45	11.16±1.41	1.82±0.12	3.55±0.33
***Protozoa***	20∶4ω6	0.26±0.02	0.19±0.15	3.71±0.30	2.44±0.45	2.97±0.10	11.93±4.10

The percentage of ^13^C allocated to a certain PLFA (plus standard errors in %) was calculated from the amount of each PLFA and total ^13^C accumulation (excess ^13^C pmol g^−1^) in all PLFAs used as biomarkers for different microbial groups.

* values are shown as percentage (%).

### Total Community RNA Extraction and Gradient Fractionation

Total nucleic acids were extracted from samples consisting of 400 mg of rhizosphere soil using the protocol of Griffiths *et al.* [29]. RNA was then enriched by treating the total nucleic acids with DNAse (Turbo DNAse; Ambion Life Technologies, Carlsbad, CA, USA) and further inspected for integrity using the Experion RNA StdSens Analysis System (Experion; Bio-Rad Laboratories Inc., Hercules, CA, USA). Total RNA was then quantified (NanoDrop ND-1000 spectrophotometer, Bio-Rad Laboratories Inc.). The resulting RNA was stored at −80°C. Following this, the ^13^C-labelled RNA was separated from unlabelled RNA by density gradient centrifugation after which it was analysed as in [30]. The ‘heavy’ RNA was thus successfully separated from the ‘light’ RNA by ultracentrifugation, as previously described [23]. We used a total of 500 ng of RNA per sample and collected 20 fractions from the density gradient after centrifugation. The fractionated RNA was then pooled into samples denoted as ‘heavy’ and ‘light’ based on the presence of nucleic acids (measured with NanoDrop) at the desired densities. The first pool contained fractions consisting of ^13^C-labelled RNA and the second unlabelled (^12^C) RNA. The ^12^C RNA from unlabelled plants was used as controls and analysed in the same way as the RNA from ^13^C-labeled plants.

### Reverse Transcription - polymerase Chain Reaction (RT-PCR)

Reverse transcription of RNA to complementary DNA (cDNA) was performed according to [21] using random hexamer primers and the Superscript II RNase H - reverse transcriptase kit (Invitrogen, Paisley, UK). The pooled fractions were successfully reverse-transcribed into cDNA and offered target amplicons suitable for PCR-DGGE as well as clone library analyses, allowing the comparison of the responder groups among the bacteria in the analysed rhizosphere samples. PCR amplification of the cDNA was performed as for DNA using the random hexamer primers. The resulting PCR-generated amplicons were subjected to further analyses.

### Denaturing Gradient Gel Electrophoresis (DGGE) Analysis

PCR-DGGE fingerprints were obtained for the domain Bacteria and for the specific classes Alphaproteobacteria and Betaproteobacteria. Aliquots of the cDNAs obtained from each sample were used for amplification of the 16S ribosomal RNA (rRNA) gene regions. Amplification was performed in a Gene Amp PCR System 2400 (Applied Biosystems), in a 50-µL reaction containing 5 ng of cDNA and 400 nmol L^−1^ of 16S rRNA gene universal primers 968F-GC and 1401R-1b [31]. To evaluate the two specific groups, an initial PCR was performed with primers specific for the Alphaproteobacteria or Betaproteobacteria [32] in combination with primer 1401R-1b [31]. The amplified PCR products were then used as templates in separate nested PCRs with primers 968F-GC and 1401R-1b [31]. DGGE analysis was conducted as described previously [33], using an Ingeny phorU2 apparatus (Ingeny International, Goes, The Netherlands). PCR products were loaded onto 6% (w/v) polyacrylamide gels in 0.5 TAE buffer (20 mM Tris-acetate, 1 mM EDTA pH 8.0). The polyacrylamide gels were made with denaturing gradients ranging from 45 to 65% (where 100% denaturant contained 7 M urea and 40% formamide). The gels were run for 16 h at 100 V and 60°C, after which they were soaked for 1 h in SYBR Green I nucleic acid staining solution (Molecular Probes, Leiden, The Netherlands) and photographed under UV light. We ran one gel per target bacterial group, allowing comparison of samples within the same gel.

The DGGE patterns were analysed using GelComparII software (Applied Maths, Sint Martens Latem, Belgium), where patterns were normalized and cross-compared. Cluster analysis of DGGE patterns was performed using UPGMA (unweighted pair group method with arithmetic mean) based on the similarity calculated by densitometric Pearson correlation [34], [35].

### DGGE Band Excision and Identification

Key DGGE bands (a total of 13 for the DGGE patterns originating from Karnico and 11 from Modena), selected according to their presence in the ‘heavy’ and absence in the ‘light’ fractions, were excised from the gels. These were re-amplified using primers 968f with GC-clamp and 1401R-1b and ran on a second DGGE gel. The bands were then re-excised from the second gel and re-amplified with primers 968f and 1401R-1b. The resulting products were cloned into the pGEM-T-Easy vector and introduced into chemically-competent *Escherichia coli* JM109 (Promega, USA) cells by transformation according to the manufacturer’s instructions. Four positive clones per band were randomly chosen for sequencing. Primers M13F and M13R were used to determine which clones contained correctly-sized inserts. Inserts were sequenced by LGC Genomics (Berlin, Germany). Sequence chromatograms were trimmed using Lucy algorithm [36] at a threshold of base quality score >20. Chimeric sequences were checked using Bellerophon v.3 at the Greengenes website (http://greengenes.lbl.gov/) and removed from the further analysis. All sequences were compared to the GenBank database using Basic Local Alignment Search Tool algorithm (BLAST) nt/nt [37], and at Ribosomal Data Project webpage (http://rdp.cme.msu.edu/), using its classification system.

### Construction of 16S rRNA Gene Clone Libraries, Phylogenetic Reconstruction and Statistical Analyses

Clone libraries were constructed using the cDNA synthesized from the 16S rRNA gene transcripts from the 5-day sampling (time at which the first peak of ^13^C accumulation in bacterial fraction was observed, Table 1). In total, eight libraries were constructed, consisting of duplicates of two fractions (i.e. ‘light’ and ‘heavy’) obtained for each of the two potato lines Karnico and Modena. The PCR conditions for amplification of bacterial 16S rRNA genes was the same as described above for the DGGE protocol, except that the primers used for PCR did not have a GC clamp. Prior to cloning, the PCR fragments obtained were purified with Wizard® SV Gel and PCR clean-up systems (Promega, USA). Purified amplicons were then ligated into the pGEM-T-Easy vector and introduced into competent *Escherichia coli* JM109 (Promega, USA) following the manufacturer's instructions. Clones containing the insert (evaluated by blue/white colony) were subjected to colony PCR with primers M13F and M13R to determine which clones contained a correctly-sized insert. Sequencing reactions were prepared and analysed by LGC Genomics (Berlin, Germany).

Prior to the analyses, all chromatograms were trimmed for quality and vector removal using the Lucy algorithm [36] at a threshold of base quality score >20 and sequence length >400 bp. The presence of chimeric sequences was evaluated using Bellerophon v.3 at the Greengenes website (http://greengenes.lbl.gov/), and chimeric sequences were removed from further analyses. The frequencies of sequences affiliated within distinct genera were first recorded based on the RDP classification system (http://rdp.cme.msu.edu/), and further confirmed by comparison to the GenBank database using BLAST.

Prior to the phylogenetic analysis, each duplicate clone library was pooled and clustered into Operational Taxonomic Units (OTUs) at 99% similarity using Mothur [38]. One representative sequence per OTU was selected close to its best-matched sequence downloaded from the GenBank database. The reconstruction of phylogenetic relationships was performed using MEGA 5.0 [39], where the evolutionary history was inferred using Neighbour-Joining [40], and evolutionary distances computed using the Kimura-2 parameter method [41]. The robustness of the branch nodes was tested using bootstrap analyses (1,000 replications).

Similarity percentage (SIMPER) calculations were conducted using PRIMER-E (version 6, PRIMER-E Ltd, Plymouth, UK) [42], based on Bray-Curtis dissimilarity, to define the groups that were primarily responsible for the differences between the cultivars. Principal components analyses (PCA), based on the relative abundance values obtained, were performed with CANOCO software (version 4.52, Wageningen, The Netherlands), to assess whether any effect of potato cultivar type could be discerned. The significance of the differences between cultivars and time-points concerning fatty acids incorporation was compared with Student’s *t-*tests and differences were considered to be significant at P<0.05.

A set of representative OTU sequences, next to those of excised and sequenced bands (all partial 16S rRNA sequences) obtained in this study are submitted to the National Center for Biotechnology Information (NCBI) database under the accession numbers JX892867 to JX892926.

## Results

### Plant Growth and Phenotype

For all replicates, both cultivars Karnico and Modena developed into healthy plants up to the EC90 stage, during the time prior to the ^13^C-CO2 pulse. Also, during the 12 days inside the growth chambers (kept for 24 h at 350 to 380 ppm of ^13^C-CO_2_), no visible signs of disease or other plant stresses were detected. Hence, the ^13^C-CO_2_ pulse did not visibly affect plant vigour in either of the two cultivars [23]. During the pulse period, the plants were shown to steadily consume the ^13^C-CO_2_ and thus integrate considerable ^13^C label in their aboveground and belowground parts [23]. There were no conspicuous differences in the amount of label captured by each of the plants.

### PLFA Biomarkers

**Figure 1:**
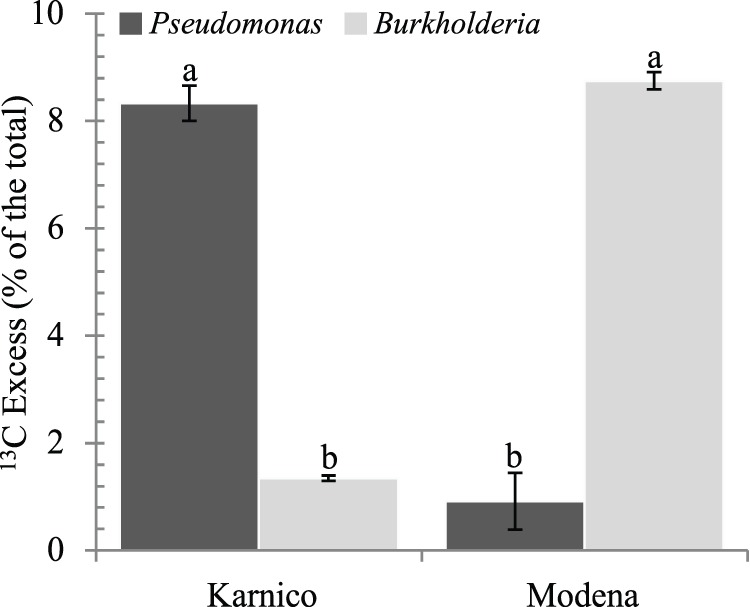
Effect of cultivar in the amount of excess ^13^C (% of the total) as measured by phospholipid fatty acid (PLFA) analyses (average values over time). The incorporation of ^13^C into the markers was calculated based on markers specific to these groups mentioned in text at three time-points. PLFAs used as indicators for the different microbial groups are given in the Materials and Methods section. Bars represent standard errors. Letters represent significant statistical differences (P<0.05) between cultivars.

Analysis of the heavy (^13^C) PLFA from the rhizosphere samples showed that bacteria had accumulated about 13±1.21% and 17±1.77% (24 h), 47±5.37% and 41±5.06% (5 days), and 30±2.81% and 57±4.39% (12 days) of the total label into fatty acids, for cultivars Karnico and Modena respectively (Table 1). This indicated highly active bacteria usurping the carbonaceous compounds released from the roots of both cultivars, as from the onset of the experiment. A significant difference between cultivars in the accumulation of fatty acids was observed only after 12 days, 30±2.81% for cv Karnico versus 57±4.39 for cv Modena (P = 0.043). Gram-positive bacteria, as evidenced by biomarkers i15∶0, a15∶0, i16∶0, a17∶0 and 10 Me16∶0, incorporated 14±0.52% to 40% ±0.29% of the label that entered the bacteria, which differed significantly between the cultivars at 24 h (P = 0.035) and 12 (P = 0.039) days. Among the Gram-positive bacteria, *Bacillus* made up between 10±3.55% and 63±5.37% of the total, whereas values of *Actinobacteria* varied between 7±1.38% and 50±22.85% of the total (except for cv Modena at 24 h which did not incorporate biomarkers for *Actinobacteria*). Whereas *Actinobacteria* were more prevalent at cv Modena (on average 31±0.75% versus 14±0.3% for cv Karnico), *Bacillus* species were more abundant in cv Karnico (on average 43±0.5 versus 38±0.3% for cv Modena) (Figure S1). In contrast, Gram-negative bacteria were the major bacterial users of the released substrate, revealing (at all time) to capture between about 53±1.14% and 78±2.78% of the total bacterial label. Here, a significant difference between the two cultivars was noticed, mainly after 24 h (78% ±1.14% in cv Modena versus 53% ±0.89% in cv Karnico, P = 0.023) and 5 days (76% ±3.70% in cv Karnico versus 57% ±2.58% in cv Modena, P = 0.038). Among the Gram-negative bacteria, *Pseudomonas* and *Burkholderia* (indicated by biomarkers cy17∶0 and cy19∶0, respectively) constituted up to 12±0.69% *Pseudomonas*) and 16±1.10% (*Burkholderia*) of the total (Figure S1). Whereas the cy19∶0 label (*Burkholderia*) was more prevalent at cultivar Modena (on average 8.75% ±0.33% versus 1.4% ±0.5% cv Karnico, P = 0.049), *Pseudomonas* (cy17∶0) were more abundant at cv Karnico (on average 8.33% ±0.16% versus 0.9% ±0.1% in cv Modena, P = 0.034) (Figure 1 and Table 1).

On a general notice, protozoa started to increase over the time of the experiment, as about up to 0.3±0.02% of the root-released ^13^C label was protozoal at 24 h, followed by 2.4±0.45% to 3.7±0.30% (5 days) and finally about 3.0±0.10%–11.9±4.10% (12 days). The latter high value was found with the Modena-12 days sample, indicating high protozoan predation of active bacteria in cv Modena at this time point.

### Structures of the Bacterial Communities in the Rhizospheres of Potato Cultivars Karnico and Modena

**Figure 2:**
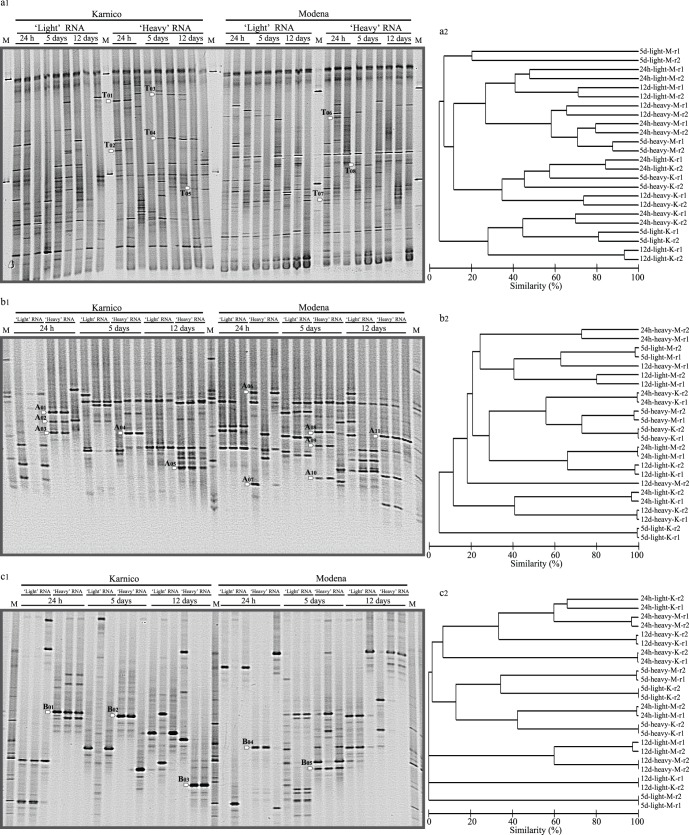
PCR-DGGE profiles of ‘light’ and ‘heavy’ fraction of labelled RNA retrieved after 24 h, 5 and 12 days of incubation, for Karnico and Modena potato cultivars: (a1) Total bacteria, (b1) Alphaproteobacteria and (c1) Betaproteobacteria community structures. White arrows indicate the position of selected bands excised from the gels. Cluster dendrograms using UPGMA based on similarity calculated by densitometric Pearson correlation analysis are shown next to each DGGE profile (a2, b2 and c2). Dendograms were generated discarding one replicate per treatment, as a few of them were considered to represent a technical outlier.

An overview of the PCR-DGGE profiles of total bacteria revealed striking levels of similarity, next to dissimilarity, across the replicates per treatment (Figure 2 a, b, c). Specifically, both in the ‘light’ and ‘heavy’ RNA derived patterns, either two or all three replicates were shown to be virtually identical. In the former case, i.e. with two virtually identical replicates, one replicate was considered to represent a technical outlier. In all further analyses, we considered the consistent patterns, i.e. focused on consistencies rather than differences, as specified in the result descriptions below.

On the basis of the selected replicates, clear differences could be observed between the patterns derived from the ‘heavy’ versus the ‘light’ RNAs, often revealing different numbers and positioning of bands (Figure 2). The ‘light’ RNA derived profiles showed diverse numbers and types of bands, with 15–50, 5–12 and 3–12 bands in the total bacterial, alpha- and betaproteobacterial patterns, respectively. The ‘heavy’ profiles encompassed 20–60, 7–16 and 4–13 bands in the total bacterial, alpha- and betaproteobacterial patterns, respectively (Figure 2 a1, b1, c1).

### Total Bacterial PCR-DGGE Profiles

Visual inspection of the bacterial PCR-DGGE banding patterns derived from cultivar Karnico revealed a total of 49 bands in the patterns from the ‘light’ fraction versus 54 in those from the ‘heavy’ fraction. The patterns from cultivar Modena showed, respectively, 43 and 49 bands in the ‘light’ and ‘heavy’ fractions (Figure 2 a1). Cluster analysis performed for the heavy RNA derived PCR-DGGE patterns indicated the formation of groups driven by cultivar as the main effector (Figure 2 a2). Analysis of selected bands (not present or present at low intensity in the light RNA derived patterns) excised from these patterns (a total of 8) revealed, for cultivar Karnico, the dominant presence of active organisms that were related to *Pseudomonas* sp. In addition, evidence was obtained for the presence of *Gluconacetobacter diazotrophicus* (24 h), *Streptococcus thermophiles, Kocuria* sp. (5 days) and *Micrococcus* sp. (12 days). A similar analysis performed for cultivar Modena revealed the dominance of organisms related to *Burkholderia* sp., next to *Corynebacterium jeikeium* (24 h) as well as an organism related to an as-yet-uncultured bacterium (5 days) (Table 2). The differential presence of *Pseudomonas* versus *Burkholderia* types at cv Karnico versus Modena was remarkable.

**Table 2:** Taxonomic affiliation of cloned 16S rRNA genes amplified from density-resolved ‘heavy’ ^13^CO_2_-incubated plant *Solanum tuberosum* cultivars Karnico and Modena.

	Cultivar/days	Closest NCBI match (accession number)/% identity
**16S-DGGE bands (bp)**		
T01 (345)	Karnico (24 h)	*Pseudomonas* sp. AB_13 16S (JQ033386)/99
T02 (395)	Karnico (24 h)	*Gluconacetobacter diazotrophicus* LMG 22174 (JF793980)/99
T03 (392)	Karnico (5 days)	*Streptococcus thermophilus* NBRC 13957 (AB680535)/99
T04 (397)	Karnico (5 days)	*Kocuria* sp. SGb392 (HQ224638)/99
T05 (394)	Karnico (12 days)	*Micrococcus* sp. S2H27 (HE814750)/99
T06 (393)	Modena (24 h)	*Burkholderia* sp. SAP53 (JN872507)/100
T07 (393)	Modena (24 h)	*Corynebacterium jeikeium* K411 (CR931997)/100
T08 (392)	Modena (5 days)	Uncultured bacterium clone ncd1056g11c1 (HM344205)/100
**Alpha-DGGE bands (bp)**		
A01 (396)	Karnico (24 h)	*Bosea thiooxidans* KB13-VS (AJ250800)/99
A02 (396)	Karnico(24 h)	Alpha proteobacterium CCBAU 45397 (HM107183)/98
A03 (396)	Karnico(24 h)	*Bradyrhizobium* sp. S3HL1 (HE814814)/99
A04 (396)	Karnico(5 days)	*Bradyrhizobium* sp. RS-46 (FM998034)/99
A05 (396)	Karnico(12 days)	*Mesorhizobium* sp. SAB10 (HQ836165)/98
A06 (395)	Modena(24 h)	Alpha proteobacterium BAC47 (EU180511)/99
A07 (395)	Modena(24 h)	*Azospirillum* sp. T2-YC6788 (GQ369056)/98
A08 (396)	Modena(5 days)	*Bradyrhizobium* sp. S3HL1 (HE814814)/98
A09 (395)	Modena(5 days)	Rhizobiales bacterium C2 16S (JQ773443)/99
A10 (395)	Modena(5 days)	*Azospirillum* sp. T2-YC6788 (GQ369056)/99
A11 (396)	Modena(12 days)	*Bradyrhizobium* sp. Wall28 (EF601950)/98
**Beta-DGGE bands (bp)**		
B01 (393)	Karnico(24 h)	*Polaromonas* sp. Cr4-12 (HM583568)/100
B02 (390)	Karnico(5 days)	*Herbaspirillum* sp. Juv924 (JN590313)/98
B03 (389)	Karnico(12 days)	Burkholderiales bacterium (FN794216) V2M6/98
B04 (375)	Modena(24 h)	*Massilia* sp. Sco-D23 (FN386766)/99
B05 (389)	Modena(5 days)	Uncultured *Burkholderia* sp. SFeB26 (JQ723618)/100

### Alphaproteobacterial PCR-DGGE Profiles

The PCR-DGGE patterns generated for the Alphaproteobacteria revealed communities that were composed of limited numbers of dominating types across all samples, in the ‘light’ and ‘heavy’ RNA derived from both cultivars, Karnico and Modena. Totals of 23 and 37 bands were found in the ‘light’ fractions, versus 28 and 27 in the ‘heavy’ ones in the patterns from cultivars Karnico and Modena, respectively (Figure 2 b1, b2). Analysis of the identities of selected differential bands (dominantly present in the ‘heavy’ RNA derived patterns and absent from the ‘light’ RNA derived patterns) revealed differences in dominating Alphaproteobacteria across the two cultivars. For cultivar Karnico, organisms related to *Bosea thiooxidans*, Alphaproteobacterium CCBAU 45397 and *Bradyrhizobium* sp. were found to dominate (24 h). At 5 days, we obtained evidence for the dominance of organisms related to *Bradyrhizobium* sp. and after 12 days for those of *Mesorhizobium* sp. (Table 2). For cultivar Modena, we observed organisms related to Alphaproteobacterium BAC47 and *Azospirillum* sp. at 24 h, *Bradyrhizobium* sp., “Rhizobiales bacterium” and *Azospirillum* sp. at 5 days and again *Bradyrhizobium* sp. at 12 days (Table 2). Hence, identifiable Alphaproteobacteria were differentially selected by cultivars Karnico and Modena, next to the commonality in the selection by these cultivars of *Bradyrhizobium* types.

### Betaproteobacterial PCR-DGGE Profiles

The PCR-DGGE patterns of the Betaproteobacteria revealed communities composed of limited numbers of dominating organisms across all samples for cultivars Karnico and Modena in the ‘light’ and ‘heavy’ RNA derived patterns. Totals of 14 and 19 bands were found in the ‘light’ fraction derived patterns, versus 19 and 20 in the ‘heavy’ fraction derived ones from cultivars Karnico and Modena, respectively (Figure 2 c1, c2). Analysis of the putative identity of five selected responder bands revealed different dominating organisms across the two cultivars. For cultivar Karnico, organisms (absent in Modena) related to *Polaromonas* sp. (24 h), *Herbaspirillum* sp. (5 days) and the family Burkholderiales (12 days) were found to dominate. For cultivar Modena, we found as dominating organisms (absent from Karnico) *Massilia* sp. (24 h) and uncultured *Burkholderia* sp. (12 days) (Table 2).

### Analysis of Bacteria Actively Involved in the Capture of 13C from Potato Cultivars Modena and Karnico

**Figure 3:**
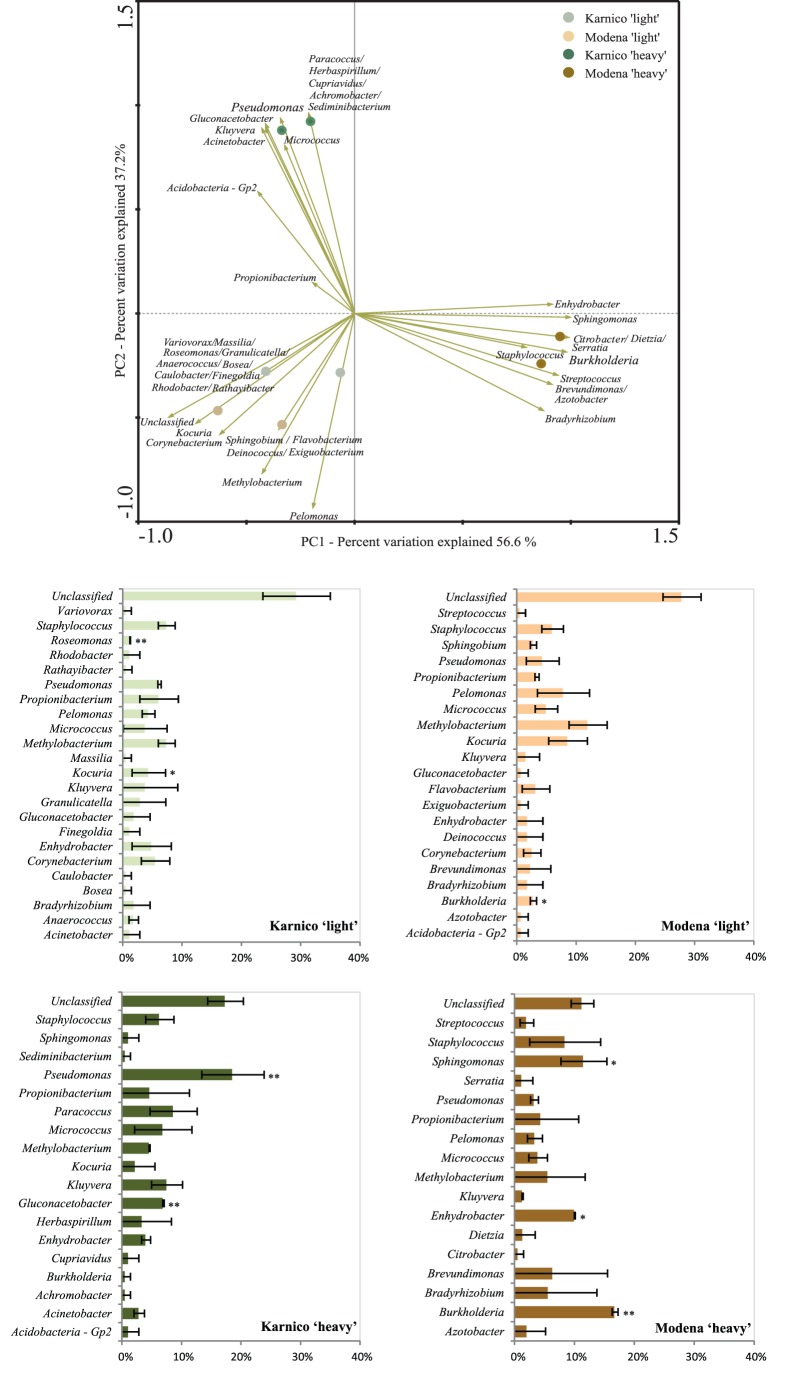
Ordination biplots generated by principal component analysis (PCA) of bacterial communities and the frequency of sequences affiliated to genera with differential occurrence in heavy fractions of RNA from each potato cultivar. Data is based on 16S rRNA gene sequences retrieved from clone libraries Karnico ‘heavy’ and Modena ‘heavy’. The eigenvalues displayed on the diagram axes refer to the percentage variation of the respective axis. Bar charts display the relative abundance of each taxonomic group considered in the analysis. The bars represent standard deviation calculated from duplicated clone libraries. * P<0.05, ** P<0.01.

In order to obtain a more thorough view of the identity of the organisms involved in the assimilation of ^13^C-labelled compounds from the two potato cultivars, we performed an analysis of the distribution of sequences from eight clone libraries (four treatments in duplicates) based on cDNA from the day-5 samples. The libraries encompassed totals of 195, 171, 199 and 156 (duplicates summed) partial sequences of the 16S rRNA gene from the Karnico ‘light’ and ’heavy’ and the Modena ‘light’ and ’heavy’ fractions, respectively. Analyses of the taxonomic affiliation of all sequences revealed that, besides the presence of low levels (on average 14%) of unclassified bacteria, the majority of all sequences could be assigned to a total of 42 different bacterial genera. Furthermore, the differential occurrence of these genera between the libraries was explored. Clearly, shifts between the ‘light’ and ‘heavy’ fraction of both cultivars could be observed when comparing the occurrence of genera in these (Figure 3). For instance, in the ‘heavy’ fraction libraries, the abundance of the genus *Pseudomonas* was significantly higher (20% ±5) in cv Karnico, when compared to cv Modena (4% ±2). On the other hand, the abundance of *Burkholderia* types was significantly higher at cv Modena (17±1) than at cv Karnico (1±1). These results were consistent with the findings by PCR-DGGE of total bacterial communities.

In addition, in order to determine the effects of plant cultivar on the root exudate activated bacteria, we cross-compared the sequence types in the ‘heavy’ fractions between the two cultivars. SIMPER analysis showed that the dissimilarity (Bray-Curtis index) between cultivars Modena and Karnico was around 47%, and that this difference occurred mostly due to the differential frequency ranges of the genera *Burkholderia* and *Pseudomonas* (together contributing to 17% of the difference), next to *Paracoccus, Gluconacetobacter* and *Sphingomonas* (Table 3), which can be indicated as highly differentially responsive genera. Specifically, the analyses supported the PCA results and suggested that the genus *Pseudomonas* (in addition to *Gluconacetobacter* and *Paracoccus)* was preferentially activated by the root-released compounds from cultivar Karnico, while the genus *Burkholderia* (next to *Moraxella*-like and *Sphingomonas)* was mostly ‘attracted’ by those from cultivar Modena (Figure 3).

**Table 3:** SIMPER analysis results displaying the top ten 99% OTUs accountable for the dissimilarity between the ‘heavy’ fractions in both potato cultivars.

Genus	Contribution (%)^a^	Mean abund^b^	Mean abund^b^
		Karnico (%)	Modena (%)
*Burkholderia*	9.28	0.54	4.10
*Pseudomonas*	7.59	4.30	1.82
*Paracoccus*	6.86	2.91	0.00
*Gluconacetobacter*	6.85	2.64	0.00
*Sphingomonas*	6.40	0.77	3.38
*Pelomonas*	4.76	0.00	1.83
*Brevundimonas*	4.67	0.00	1.79
*Acinetobacter*	4.40	1.70	0.00
*Bradyrhizobium*	4.37	0.00	1.69
*Kluyvera*	4.06	2.73	1.16

a Contribution of OTUs to overall dissimilarity between groups.

b Average abundance of OTUs in each group.

**Figure 4:**
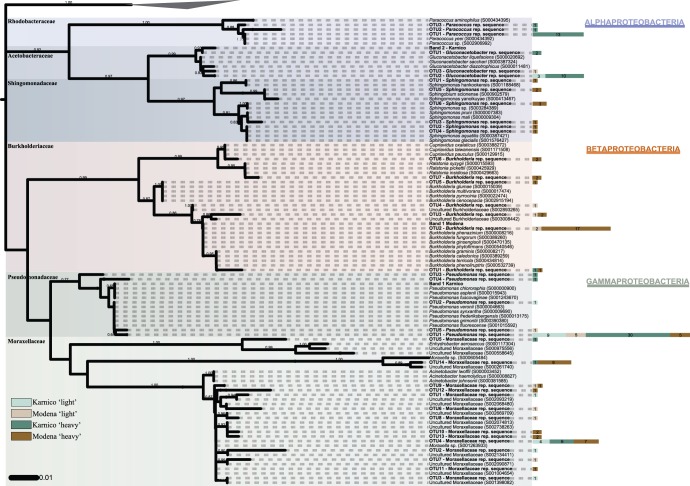
Phylogenetic reconstruction of bacterial 16S rRNA gene sequences retrieved for the Karnico ‘light’ and ‘heavy’ and Modena ‘light’ and ‘heavy’ clone libraries. Bootstrap values (1,000 repetitions) above 50% are indicated next to the tree branches. The tree displays one representative sequence per OTU and retrieved bands from DGGE (both in bold). Type and non-type sequences closed affiliated to OTU representative (rep.) sequences were retrieved from RDP database to enrich phylogenetic accuracy. A single OTU embraces all clone sequences sharing at least 99% similarity. Side bars next to OTU rep. sequences labels indicate the number of clones belonging to the correspondent OTU for each of the libraries. There were a total of 729 nucleotides position in the final dataset, and sequences belonging to the Cyanobacteria phylum were used as outgroup.

Another issue addressed by the analyses was the differential occurrence of bacterial types within each group pinpointed as a differential responder for each cultivar. Thus, we confirmed the narrow affiliation of sequences within the genera, except for the group classified as *Moraxella*-related species (Figure 4). While other groups generated OTU numbers between 3 and 7 (based on 99% similarity for clustering), the group denoted *Moraxella*-related (Moraxellaceae family) was found to encompass 14 OTUs, from the 38 sequences that were initially allocated to this genus. The 14 OTUs were diverse, mainly clustering with *Moraxella* spp. and uncultured Moraxellaceae. Considering the other groups, the abundant OTUs were mainly affiliated to known species. For instance, *Pseudomonas fluorescens* was repeatedly found, whereas within the genus *Burkholderia,* members of the *Burkholderia cepacia* complex were dominant. An abundance of sequences related to *G. diazotrophicus* was also clearly indicated, mostly in cv Karnico (Figure 4). Overall, the phylogenetic reconstruction revealed the presence of a few highly abundant OTUs (e.g. ‘OTU1– *Pseudomonas* rep. sequence’ containing 49 sequences), next to numerous other OTUs composed of only a few sequences each. The distribution of these sequences in a rank can be observed in detail in Figure 4.

## Discussion

In the current study, we assessed the impact of ^13^C-labelled carbonaceous compounds released into the rhizosphere on the local bacterial communities associated with two potato cultivars, i.e. the parental line Karnico and the GM derivative Modena, using stable isotope probing [14]. Next to assessing effects on total bacteria, we placed a special emphasis on selected bacteria with relevance for rhizosphere function, i.e. members of the Alpha- and Betaproteobacteria.

The PLFA-SIP data clearly indicated that, next to fungi [23], bacteria are among the first responders to the carbonaceous compounds that are released from potato roots (detected after 24 h), being their apparent roles enhanced and very dominant after 5 days, with a slow decline after 12 days. Among the responders, Gram-negative bacteria were found to play major roles, but a role for Gram-positive bacteria cannot be ruled out. Furthermore, on the basis of the PLFA data, we found evidence for an increased role of protozoan predation on bacteria in the GM cultivar Modena, as opposed to the relative absence of this phenomenon in cv Karnico. We do not quite understand the trigger of this difference, but it is a truly remarkable observation that warrants further studies.

The Gram-negative bacteria *Pseudomonas* and *Burkholderia* spp. have been recognized as key inhabitants of the rhizosphere [3], [27]. More specifically, previous work has indeed provided solid evidence for both *Pseudomonas* and *Burkholderia* spp. as inhabitants of the potato rhizosphere [43], [44], [45]. Using the specific fatty acids cy-17 and cy-19 (see Materials and Methods), we detected these genera in the active communities in the rhizospheres of both cultivars Karnico and Modena. Remarkably, we found differences in the relative proportions of *Pseudomonas* versus *Burkholderia* based on both the PFLA and PCR-DGGE data. Also, similar differential occurrence was found in the clone libraries (‘heavy fractions’) (Figure 3). On the basis of these observations, we conclude that cultivars Karnico and Modena exerted differential selective pressures on the root-associated communities, resulting in a significantly raised relative abundance of *Pseudomonas* in one, and of *Burkholderia* in the other cultivar. We ignore whether these different genera, both known for their metabolic flexibility and avidity in capturing a plethora of carbonaceous compounds from plant roots, serve similar or different ecological roles and thus whether we are observing an example of niche exclusion. We also cannot affirm, at this point in time, to what extent this difference is a particular characteristic brought about into cv Modena by the genetic modification event or whether it falls within the normal operating range of potato cropping. Finally, it would be interesting to ascertain what the wider implications, e.g. in terms of plant health or growth stimulation, of these differences are.

Moreover, on the basis of our RNA-SIP data, we found great dynamism in the root-responsive bacterial communities, reflected in the dynamic shifts in the bacterial communities over time at the two cultivars. Within the confines of the method applied, our data clearly showed differences between the PCR-DGGE profiles representing the ‘heavy’ and the ‘light’ RNA fractions at 24 h, 5 and 12 days following the ^13^C-CO_2_ pulse (Figure 2 a, b and c). This indicated that particular fractions of the total bacterial communities had been activated from the background of the extant bacteriota. Remarkably, major differences were found in the PCR-DGGE profiles compared across the two cultivars, possibly indicating the “rise and fall of bacterial empires” in connection with differential exudation patterns between the two plant cultivars [46]. Recently, Smyth *et al.* [47] suggested that plants like bean, wheat and sugar beet, in the course of their growth, affect the structure and activity of the rhizosphere microbiota in a dynamic fashion. Here, we provide evidence for the contention that the bacteria activated by cultivar Modena are different from those by cultivar Karnico. Overall, our data corroborate those of Rasche *et al*. [48], who found differences in primary endophytic potato colonizers when comparing plants with distinct genotypes.

Here, the phylogeny-based analyses made on the basis of the clone libraries confirmed the differential composition of bacterial communities in the ‘heavy’ and ‘light’ fractions of the RNA. Moreover, this approach identified six genera as the most prominent differential responders between cultivars Modena and Karnico. Thus, *Pseudomonas,* next to *Gluconacetobacter* and *Paracoccus,* was preferentially active in the rhizosphere of Karnico, whereas *Burkholderia* (next to *Sphingomonas* and *Moraxella*-related species) was so in the rhizosphere of cultivar Modena. These data are relevant, as all detected active genera belong to the major responding bacterial group found on the basis of the PCR-DGGE and PFLA data, i.e. the Gram-negatives/Proteobacteria. Such organisms can become dominant in soils, as well as in associations with plant roots in the rhizosphere, some of which are beneficial [9], [49], [50], [51].

With respect to the groups that were preferentially selected by cultivar Karnico, the genus *Pseudomonas* is known to harbour primary responders to root exudates, such as found in maize, wheat and colza [19], [52]. Besides, pseudomonads are often beneficial to plants due to their pathogen-suppressive and/or plant growth promoting (PGPR) action [53], [54]. The occurrence of particular *Pseudomonas* spp. can be strongly affected by the factors sampling site, time and plant type [55]. Recently, Andreote *et al* [56] revealed the endophytic occurrence of *Pseudomonas* sp. in potato, indicating a tight association with this host plant. This was consistent with previous findings of endophytic pseudomonads by van Overbeek and van Elsas [4] and Rasche *et al.* [22].

Moreover, the second selected bacterial genus, *Gluconacetobacter,* has been described as plant-associated and nitrogen-fixing, with a remarkable role in gramineous and other non-nodulating plants [57]. The attraction of these organisms to the rhizosphere can be driven by root exudates rich in carbon, which may support biological nitrogen fixation in order to balance the carbon:nitrogen ratio. In addition, the occurrence of the genus *Paracoccus* can be explained following the same line of reasoning, since this genus has been described as presenting a high metabolic flexibility, degrading different carbon sources and pollutants in soil, as denitrifiers and also as sulphur-oxidizing bacteria [58], [59].

Considering the Modena-selected genera, *Burkholderia* has recently been reported to be important in the potato rhizosphere [9]. It also made up over 3.5% of the total microbiota in the maize rhizosphere and was a nitrogen-fixing symbiont of *Mimosa* spp. [60], [61]. Some *Burkholderia* types can act as plant growth promoters, by producing siderophores and phytohormones [45] or inhibiting the development of plant pathogens [44]. *Burkholderia* is also responsive to the differential use of soil, being a major driver of diversity in the bacterial community [62]. The sequences affiliated with *Moraxella*-related species were diverse, affiliated to unclassified sequences within the Moraxellaceae. The genus *Moraxella* has been poorly studied in association with plants, but sequences of *Moraxella* spp. were recently obtained from DGGE bands in long-term *in vitro* cultivated plants [63], possibly indicating their role as endophytes. *Sphingomonas* has been described in association with soil affected by the release of carbonaceous compounds originating from plant roots [64] or from fungal hyphae, being an active member of the mycosphere [65]. This clearly indicates that such organisms may represent sensors of variation in the chemical profile of root exudates due to modifications in plant metabolism.

In our study, the aforementioned groups responded differentially to the cultivars, indicating that the association of these organisms with potato plants is sensitive to changes in plant metabolism such as caused by the genetic modification event in cultivar Modena. In previous work, several authors indicated that distinct bacterial communities might develop at different cultivars of potato, in the aerial [22] or root parts [7], [10]. We here extend this observation, to indicate that plants also drive the active fractions of the microbial communities of potato rhizosphere in a cultivar-specific manner. Considering the rhizosphere a plant shield against pathogens and an active tissue for nutritional supplementation of plants, we posit here that plant genetic modification can modulate the composition of the rhizosphere microbiome. It is difficult to pinpoint the exact mechanisms behind the selective process that we monitored, as for more detailed analysis knowledge of the precise dynamism in the exudate compositions will be required, which is an inherently difficult task [66]. A possible explanation for the differences can be the differential chemical composition of root-released carbonaceous compounds between cultivars Modena and Karnico, towards a shift in carbon metabolism in the Modena cultivar, more specifically a shift in the amylopectin/amylose ratio. Such shift might lead to key bacterial types responsive to plant roots (i.e. *Pseudomonas* and *Burkholderia* types) being selected in a plant cultivar type specific manner. However, given potential collateral secondary effects, we cannot explicitly link the differences found to the GM event in cultivar Modena.

## Supporting Information

Figure S1 **Effect of cultivar in the amount of excess ^13^C (% of the total) in different microbial groups as measured by phospholipid fatty acid (PLFA) analyses, (a)** ***Burkholderia*** **and** ***Pseudomonas*** **, and (b)** ***Bacillus*** **and** ***Actinobacteria*** **.** The incorporation of ^13^C into the markers was calculated based on markers specific to these groups at three time-points, after 24 h, 5 and 12 days. PLFAs used as indicators for the different microbial groups are given in the Materials and Methods section. Bars represent standard errors.
(EPS)

## References

[1] Duineveld BM, Rosado AS, van Elsas JD, van Veen JA (1998) Analysis of the dynamics of bacterial communities in the rhizosphere of the *chrysanthemum* via denaturing gradient gel electrophoresis and substrate utilization patterns. Appl Environ Microb 64: 4950–4957. 10.1128/aem.64.12.4950-4957.1998 PMC90948 9835588

[2] Dini-Andreote F, van Elsas JD (2013) Back to the basics: the need for ecophysiological insights to enhance our understanding of microbial behaviour in the rhizosphere. Plant Soil *In Press.* doi ––10.1007/s11104–013–1687-z.

[3] Van Overbeek LS, van Elsas JD (2008) Effects of plant genotype and growth stage on the structure of bacterial communities associated with potato (*Solanum tuberosum* L.). FEMS Microbiol Ecol 64: 283–96. 18355298 10.1111/j.1574-6941.2008.00469.x

[4] Garbeva P, van Veen JA, van Elsas JD (2004) Microbial diversity in soil: selection of the microbial populations by plant and soil type and implementations for disease suppressivenss. Annu Rev Phytopathol 42: 243–270. 15283667 10.1146/annurev.phyto.42.012604.135455

[5] De Bruijn FJ (2013) Molecular microbial ecology of the rhizosphere. Ed. De Bruijn FJ, John Wiley & Sons, Incorporated, ISBN 1118296176, 746.

[6] De Vetten N, Wolters A, Raemakers K, van der Meer I, ter Stege R, et al (2003) A transformation method for obtaining marker-free plants of a cross-pollinating and vegetatively propagated crop. Nature Biotech 21: 439–442. 10.1038/nbt801 12627169

[7] İnceoğlu Ö, Al-Soud WA, Salles JF, Semenov AV, van Elsas JD (2011) Comparative analysis of bacterial communities in a potato field as determined by pyrosequencing. PLoS ONE 6(8): e23321 doi:10.1371/journal.pone.0023321 21886785 10.1371/journal.pone.0023321 PMC3158761

[8] Dias ACF, Hoogwout EF, Pereira e Silva MC, Salles JF, van Overbeek LS, et al (2012) Potato cultivar type affects the structure of ammonia oxidizer communities in field soil under potato beyond the rhizosphere. Soil Biol Biochem 50: 85–95.

[9] Gschwendtner S, Esperschütz J, Buegger F, Reichmann M, Müller M, et al (2011) Effects of genetically modified starch metabolism in potato plants on photosynthate fluxes into the rhizosphere and on microbial degraders of root exudates. FEMS Microbiol Ecol 76: 564–75. 21348886 10.1111/j.1574-6941.2011.01073.x

[10] İnceoğlu Ö, Salles JF, Van Overbeek L, Van Elsas JD (2010) Effect of plant genotype and growth stage on the ß-proteobacterial community associated with different potato cultivars in two fields. Appl Environ Microb 76: 3675–3684. 10.1128/AEM.00040-10 PMC2876460 20363788

[11] Lynch JM (1994) The rhizosphere - form and function. Appl Soil Ecol 1: 193–198.

[12] Bardgett RD, Mawdsley JL, Edwards S, Hobbs PJ, Rodwell JS, et al (1999) Plant species and nitrogen effects on soil biological properties of temperate upland grasslands. Funct Ecol 13: 650–660.

[13] Kuzyakov Y, Domanski G (2000) Carbon input by plants into the soil. Review. J Plant Nutr Soil Sc -Zeitschrift Fur Pflanzenernahrung Und Bodenkunde 163: 421–431.

[14] Prosser JI, Rangel-Castro JI, Killham K (2006) Studying plant microbe interactions using stable isotope technologies. Curr Opin Biotech 17: 98–102. 16413769 10.1016/j.copbio.2006.01.001

[15] Bernard L, Mougel C, Maron PA, Nowak V, Lévêque J, et al (2007) Dynamics and identification of soil microbial populations actively assimilating carbon from ^13^C-labelled wheat residue as estimated by DNA- and RNA-SIP techniques. Environ Microbiol 9: 752–764. 17298374 10.1111/j.1462-2920.2006.01197.x

[16] Semenov AV, Pereira e Silva MC, Szturc-Koestsier AE, Schmittb H, Salles JF, et al (2012) Impact of incorporated fresh ^13^C potato tissues on the bacterial and fungal community composition of soil. Soil Biol Biochem 49: 88–95.

[17] Vandenkoornhuyse P, Mahe S, Ineson P, Staddon P, Ostle N, et al (2007) Active root-inhabiting microbes identified by rapid incorporation of plant-derived carbon into RNA. P Natl Acad Sci USA. 43: 16970–16975. 10.1073/pnas.0705902104 PMC2040396 17939995

[18] Haichar Fel Z, Roncato MA, Achouak W (2012) Stable isotope probing of bacterial community structure and gene expression in the rhizosphere of *Arabidopsi*s *thaliana* . FEMS Microbiol Ecol 81: 291–302. 22385286 10.1111/j.1574-6941.2012.01345.x

[19] Haichar FZ, Marol C, Berge O, Rangel-Castro JI, Prosser JI, et al (2008) Plant host habitat and root exudates shape soil bacterial community structure. ISME J 2: 1221–1230. 18754043 10.1038/ismej.2008.80

[20] Lu Y, Abraham WR. Conrad R (2006) Spatial variation of active microbiota in the rice rhizosphere revealed by in situ stable isotope probing of phospholipid fatty acids. Environ Microbiol 9: 474–81. 10.1111/j.1462-2920.2006.01164.x 17222145

[21] Rangel-Castro JI, Killham K, Ostle N, Nicol GW, Anderson IC, et al (2005) Stable isotope probing analysis of the influence of liming on root exudate utilization by soil microorganisms. Environ Microbiol 7: 828–838. 15892702 10.1111/j.1462-2920.2005.00756.x

[22] Rasche F, Hödl V, Poll C, Kandeler E, Gerzabek MH, et al (2006) Rhizosphere bacteria affected by transgenic potatoes with antibacterial activities compared with the effects of soil, wild-type potatoes, vegetation stage and pathogen exposure. FEMS Microbiol Ecol 56: 219–235. 16629752 10.1111/j.1574-6941.2005.00027.x

[23] Hannula SE, de Boer W, van Veen JA (2012) ^13^C pulse-labeling assessment of the community structure of active fungi in the rhizosphere of a genetically starch-modified potato (*Solanum tuberosum*) cultivar and its parental isoline. New Phytol 194: 784–799. 22413848 10.1111/j.1469-8137.2012.04089.x

[24] Hannula SE, de Boer W, van Veen JA (2010) In situ dynamics of soil fungal communities under different genotypes of potato, including a genetically modified cultivar. Soil Biol Biochem 42: 2211–2223.

[25] Boschker HTS (2004) Linking microbial community structure and functioning: Stable isotope (13C) labeling in combination with PLFA analysis, p.1673–1688. In GA Kowalchuk, FJ de Bruijn, IM Head, AD Akkermans, and JD van Elsas [eds.], Molecular microbial ecology manual II. Kluwer Academic.

[26] Mauclaire L, Pelz O, Thullner M, Abraham WR, Zeyer J (2003) Assimilation of toluene carbon along a bacteria-protist food chain determined by 13C-enrichment of biomarker fatty acids. J Microbiol Methods 55: 635–649. 14607407 10.1016/s0167-7012(03)00205-7

[27] Drigo B, Pijla AS, Duytsc H, Kielak AN, Gampera HA, et al (2010) Shifting carbon flow from roots into associated microbial communities in response to elevated atmospheric CO_2_ . P Natl Acad Sci USA 107: 10938–10942. 10.1073/pnas.0912421107 PMC2890735 20534474

[28] Frostegård A, Tunlid A, Bååth E (1993) Phospholipid fatty-acid composition, biomass and activity of microbial communities from 2 soil types experimentally exposed to different heavy-metals. Appl Environ Microbiol 59: 3605–3617. 16349080 10.1128/aem.59.11.3605-3617.1993 PMC182506

[29] Griffiths RI, Whiteley AS, O’Donnell AG, Bailey MJ (2000) Rapid method for coextraction of DNA and RNA from natural environments for analysis of ribosomal DNA- and rRNA-based microbial community composition. Appl Environ Microb 66: 5488–5491. 10.1128/aem.66.12.5488-5491.2000 PMC92488 11097934

[30] Manefield M, Whiteley AS, Griffiths RI, Bailey MJ (2002) RNA stable isotope probing, a novel means of linking microbial community function to phylogeny. Appl Environ Microb 68: 5367–5373. 10.1128/AEM.68.11.5367-5373.2002 PMC129944 12406726

[31] Brons JK, Van Elsas JD (2008) Analysis of bacterial communities in soil by use of denaturing gradient gel electrophoresis and clone libraries, as influenced by different reverse primers. Appl Environ Microb 74: 2717–2727. 10.1128/AEM.02195-07 PMC2394888 18310425

[32] Gomes NCM, Heuer H, Schonfeld J, Costa R, Mendonca-Hagler L, et al (2001) Bacterial diversity of the rhizosphere of maize (*Zea mays*) grown in tropical soil studied by temperature gradient gel electrophoresis. Plant Soil 232: 167–180.

[33] Muyzer G, de Waal EC, Uitterlinden AG (1993) Profiling of complex microbial populations by denaturing gradient gel electrophoresis analysis of polymerase chain reaction amplified genes coding for 16S rRNA. Appl Environ Microb 59: 695–700. 10.1128/aem.59.3.695-700.1993 PMC202176 7683183

[34] Kropf S (2004) Nonparametric multiple test procedures with data-driven order of hypotheses and with weighted hypotheses. J Statis Plann Inf 125: 31–47.

[35] Rademaker JL, de Bruijn AF (1999) Molecular microbial ecology manual. In van Elsas JD, Akkermans ADL and de Bruijn AF, Eds. Molecular Microbial Ecology Manual. Dordrecht, The Netherlands: Kluwer Academic Publishers, 1–33.

[36] Chou HH, Holmes HM (2001) DNA sequence quality trimming and vector removal. Bionformatics. 17: 1093–1104. 10.1093/bioinformatics/17.12.1093 11751217

[37] Altschul SF, Madden TL, Schaffer AA, Zhang J, Zhang Z, et al (1997) Gapped BLAST and PSI-BLAST: A new generation of protein database search programs. Nucleic. Acids Res 25: 3389–3402. 10.1093/nar/25.17.3389 PMC146917 9254694

[38] Schloss PD, Westcott SL, Ryabin T, Hall JR, Hartmann M, et al (2009) Introducing mothur: open-source, plataform-independent, community-supported software for describing and comparing microbial communities. Appl Environ Microb 75: 7537–7541. 10.1128/AEM.01541-09 PMC2786419 19801464

[39] Tamura K, Peterson D, Peterson N, Stecher G, Nei M, et al (2011) MEGA5: Molecular Evolutionary Genetics Analysis using maximum likelihood, evolutionary distance, and maximum parsimony methods. Mol Biol Evol 28: 2731–2739. 21546353 10.1093/molbev/msr121 PMC3203626

[40] Saitou N, Nei M (1987) The neighbor-joining method–a new method for reconstructing phylogenetic trees. Mol Biol Evol 4: 406–425. 3447015 10.1093/oxfordjournals.molbev.a040454

[41] Kimura M (1980) A simple method for estimating evolutionary rates of base substitutions through comparative studies of nucleotide sequences. J Mol Evol (16) 111–120. 10.1007/BF01731581 7463489

[42] Clarke KR, Gorley RN (2006) PRIMER v6: User Manual/Tutorial. PRIMER-E, Plymouth.

[43] Costa R, Gomes NC, Krögerrecklenfort E, Opelt K, Berg G, et al (2007) Pseudomonas community structure and antagonistic potential in the rhizosphere: insights gained by combining phylogenetic and functional gene-based analyses. Environ Microbiol 9: 2260–2273. 17686023 10.1111/j.1462-2920.2007.01340.x

[44] Mendes R, Pizzirani-Kleiner AA, Araújo WL, Raaijmakers JM (2007) Diversity of cultivated endophytic bacteria from sugarcane: genetic and biochemical characterization of *Burkholderia cepacia* complex isolates. Appl Environ Microbiol 73: 7259–7267. 17905875 10.1128/AEM.01222-07 PMC2168197

[45] Luvizotto DM, Marcon J, Andreote FD, Dini-Andreote F, Neves AAC, et al (2010) Genetic diversity and plant-growth related features of *Burkholderia* spp. from sugarcane roots. World J Microbiol Biotechnol 26: 1829–1836.

[46] Hardoim PR, Hardoim CCP, van Overbeek LS, van Elsas JD (2012) Dynamics of seed-borne rice endophytes on early plant growth stages. PLoS ONE 7(2): e30438 doi:10.1371/journal.pone.0030438 22363438 10.1371/journal.pone.0030438 PMC3281832

[47] Smyth EM, McCarthy J, Nevin R, Khan MR, Dow JM, et al (2011) *In vitro* analyses are not reliable predictors of the plant growth promotion capability of bacteria; a *Pseudomonas fluorescens* strain that promotes the growth and yield of wheat J Appl Microbiol. 111: 683–692. 10.1111/j.1365-2672.2011.05079.x 21672102

[48] Rasche F, Lueders T, Schloter M, Schaefer S, Buegger F, et al (2009) DNA-based stable isotope probing enables the identification of active bacterial endophytes in potatoes New Phytol. 181: 802–807. 10.1111/j.1469-8137.2008.02744.x 19140937

[49] Dalmastri C (1999) Soil type and maize cultivar affect the genetic diversity of maize root-associated *Burkholderia cepacia* populations. Microbial Ecol 38: 273–284. 10.1007/s002489900177 10541789

[50] Sessitsch AT, Coenye AV, Sturz P, Vandamme E, Barka A, et al (2005) *Burkholderia phytofirmans* sp. nov., a novel plant-associated bacterium with plant-beneficial properties. Int J Syst Evol Micr 55: 1187–1192. 10.1099/ijs.0.63149-0 15879253

[51] Ramette A, LiPuma JJ, Tiedje JM (2005) Species abundance and diversity of *Burkholderia cepacia* complex in the environment. Appl Environ Microb 71: 1193–1201. 10.1128/AEM.71.3.1193-1201.2005 PMC1065178 15746318

[52] Dohrmann A, Tebbe CC (2005) Effect of elevated tropospheric ozone on the structure of bacterial communities inhabiting the rhizosphere of herbaceous plants native to Germany. Appl Environ Microb 71: 7750–7758. 10.1128/AEM.71.12.7750-7758.2005 PMC1317375 16332747

[53] Haas D, Défago G (2005) Biological control of soil-borne pathogens by *fluorescent pseudomonads* . Nat Rev Microbiol 3: 307–319. 15759041 10.1038/nrmicro1129

[54] Neal AL, Ahmad S, Gordon-Weeks R, Ton J (2012) Benzoxazinoids in root exudates of maize attract *Pseudomonas putida* to the rhizosphere. PLoS ONE 7(4): e35498 doi:10.1371/journal.pone.0035498 22545111 10.1371/journal.pone.0035498 PMC3335876

[55] Costa R, Gotz M, Mrotzek N, Lottmann J, Berg G, et al (2006) Effects of site and plant species on rhizosphere community structure as revealed by molecular analysis of microbial guilds. FEMS Microbiol Ecol 56: 236–249. 16629753 10.1111/j.1574-6941.2005.00026.x

[56] Andreote FD, Araújo WL, Azevedo JL, Van Elsas JD, Van Overbeek L (2009) Endophytic colonization of potato (*Solanum tuberosum* L.) by a novel competent bacterial endophyte, *Pseudomonas putida* strain P9, and the effect on associated bacterial communities. Appl Environ Microb 75: 3396–406. 10.1128/AEM.00491-09 PMC2687292 19329656

[57] Baldani JI, Caruso L, Baldani VLD, Goi SR, Döbereiner J (1997) Recent advances in BNF with non-legume plants. Soil Biol Biochem 29: 911–922.

[58] Kumar R, Singh B, Gupta VK (2012) Biodegradation of fipronil by *Paracoccus* sp. in different types of soil. Bul Environ Contam Toxicol 88: 781–787. 10.1007/s00128-012-0578-y 22371192

[59] Ghosh W, Mandal S, Roy P (2006) *Paracoccus bengalensis* sp. nov., a novel sulfur-oxidizing chemolithoautotroph from the rhizospheric soil of an Indian tropical leguminous plant. Syst Appl Microbiol 29: 396–403. 16824961 10.1016/j.syapm.2005.10.004

[60] Di Cello F, Bevivino A, Chiarini L, Fani R, Paffetti D, et al (1997) Biodiversity of a *Burkholderia cepacia* population isolated from the maize rhizosphere at different plant growth stages. Appl Environ Microb 63: 4485–4493. 10.1128/aem.63.11.4485-4493.1997 PMC168767 9361434

[61] Elliott GN, Chen WM, Chou JH, Wang HC, Sheu SY, et al (2007) *Burkholderia phymatum* is a highly effective nitrogen-fixing symbiont of *Mimosa* spp. and fixes nitrogen ex planta. New Phytol 173: 168–180. 17176403 10.1111/j.1469-8137.2006.01894.x

[62] Salles JF, De Souza FA, Van Elsas JD (2002) Molecular method to assess the diversity of *Burkholderia* species in environmental samples. Appl Environ Microb 68: 1595–1603. 10.1128/AEM.68.4.1595-1603.2002 PMC123827 11916673

[63] Almeida CV, Andreote FD, Yara R, Tanaka FAO, Azevedo JL, et al (2009) Bacteriosomes in axenic plants: endophytes as stable endosymbionts. World J Microbiol Biotech 25: 1757–1764.

[64] Ogita N, Hashidoko Y, Limin SH, Tahara S (2006) Linear 3-hydroxybutyrate tetramer (HB4) produced by *Sphingomonas* sp. is characterized as a growth promoting factor for some rhizomicrofloral composers. Biosci Biotech Bioch 70: 2325–2329. 10.1271/bbb.60299 16960351

[65] Boersma FGH, Warmink JA, Andreote FA, van Elsas JD (2009) Selection of Sphingomonadaceae at the base of *Laccaria proxima* and *Russula exalbicans* fruiting bodies. Appl Environ Microb 75: 1979–1989. 10.1128/AEM.02489-08 PMC2663213 19181827

[66] Gleixner G, Schmidt HL (1998) On-line determination of group specific isotope ratios in model compounds and acquatic humic substances by coupling pyrolysis to GC-C-IRMS. In: Stankiewics BA, van Berger PF (eds) 214^th^ National Meeting of the American Chemical Society. American Chemical Society, Las Vegas, 34–45.

